# Inactivation of *FBXW7/hCDC4-β *expression by promoter hypermethylation is associated with favorable prognosis in primary breast cancer

**DOI:** 10.1186/bcr2788

**Published:** 2010-12-01

**Authors:** Shahab Akhoondi, Linda Lindström, Martin Widschwendter, Martin Corcoran, Jonas Bergh, Charles Spruck, Dan Grandér, Olle Sangfelt

**Affiliations:** 1 Departments of Cell and Molecular Biology, Karolinska Institute, Berzelius väg 35, Box 285 S-17177, Stockholm, Sweden; 2 Departments of Oncology-Pathology, Cancer Center Karolinska, Radiumhemmet, Karolinska Institute and Hospital, S-17176, Stockholm, Sweden; 3 Department of Medical Oncology, Christie Hospital, Manchester University and Christie Hospital, Wilmslow Road, Manchester, M20 4BX, UK; 4 Department of Gynecological Oncology, Institute for Women's Health, University College London, EGA Hospital, 2nd Floor Huntley Street, London, WC1E 6DH, UK; 5 Sanford-Burnham Medical Research Institute, 10901 North Torrey Pines Road, La Jolla, CA 92037, USA

## Abstract

**Introduction:**

Mutational inactivation of the *FBXW7/hCDC4 *tumor suppressor gene (TSG) is common in many cancer types, but infrequent in breast cancers. This study investigates the presence and impact of *FBXW7/hCDC4 *promoter methylation in breast cancer.

**Methods:**

*FBXW7/hCDC4-β *expression and promoter methylation was assessed in 161 tumors from two independent breast cancer cohorts. Associations between methylation status and clinicopathologic characteristics were assessed by Fisher's exact test. Survival was analyzed using the Kaplan-Meier method in addition to modeling the risk by use of a multivariate proportional hazard (Cox) model adjusting for possible confounders of survival.

**Results:**

Methylation of the promoter and loss of mRNA expression was found both in cell lines and primary tumors (43% and 51%, respectively). Using Cox modeling, a trend was found towards decreased hazard ratio (HR) for death in women with methylation of *FBXW7/hCDC4-β *in both cohorts (HR 0.53 (95% CI 0.23 to 1.23) and HR 0.50 (95% CI 0.23 to 1.08), respectively), despite an association between methylation and high-grade tumors (*P *= 0.017). Interestingly, in subgroups of patients whose tumors are p53 mutated or lymph-node positive, promoter methylation identified patients with significantly improved survival (*P *= 0.048 and *P *= 0.017, respectively).

**Conclusions:**

We demonstrate an alternative mechanism for inactivation of the TSG *FBXW7/hCDC4*, namely promoter specific methylation. Importantly, in breast cancer, methylation of *FBXW7/hCDC4-β *is related to favorable prognosis despite its association with poorly differentiated tumors. Future work may define whether *FBXW7/hCDC4 *methylation is a biomarker of the response to chemotherapy and a target for epigenetic modulation therapy.

## Introduction

The F-box protein Fbxw7/hCdc4 is the substrate specificity component of the SCF^Fbxw7/hCdc4 ^ubiquitin ligase. SCF^Fbxw7/hCdc4 ^is responsible for the targeted ubiquitylation and subsequent proteasomal degradation of an array of oncoproteins that plays a critical role in oncogenesis, such as cyclin E, c-Myc, Notch, and c-Jun among others [1,2]. Substrate recognition is tightly regulated by phosphorylation of specific motifs in the target proteins called Cdc4 phosphodegrons (CPDs) [3]. In line with its suppressive function on oncoproteins, SCF^Fbxw7/hCdc4 ^is inactivated by mutations in various tumor types [4]. The majority of the mutations identified in Fbxw7/hCdc4 in cancer specimens are missense mutations in the binding pocket of Fbxw7/hCdc4 that prevent its interaction with the phosphorylated CPD motif in the target proteins [4,5].

The tumor suppressor function of Fbxw7/hCdc4 is further underscored by frequent deletions of its locus at chromosome 4q31, occurring in more than 30% of all neoplasms [6]. Furthermore, targeted disruption of the *Fbxw7/hCdc4 *gene has been shown to result in enhanced genomic instability [7], a hallmark of cancer cells. Studies in mice additionally support a tumor suppressive activity of Fbxw7/hCdc4. Conditional inactivation of Fbxw7/hCdc4 in the T-cell lineage of mice promoted the development of thymic lymphomas [8] and loss of one Fbxw7/hCdc4 allele was shown to accelerate tumor development in p53-heterozygous (p53+/-) mice [9].

Little is known about the transcriptional regulation of Fbxw7/hCdc4, but it has been shown to be a target of p53 activation [10], establishing a direct link between these two tumor suppressor genes (TSGs).

Three different *Fbxw7/hCdc4 *isoforms (α, β and γ) have been identified in mammals [11]. Each isoform creates proteins with identical substrate interaction domains and a shared F-box motif linking to the common core ligase components (Skp1-Cul1-Roc1), but encode unique N-terminal regions that localize each isoform to specific subcellular compartments [1,2]. Each isoform is believed to possess its own promoter, which could be differentially regulated in a cell type-specific manner. Furthermore, isoform specific interactions with several accessory proteins have been reported [12,13]. Importantly, mutations in specific isoforms have also been identified in cancers, further strengthening the notion of non-redundant functions for the three different Fbxw7/hCdc4 isoforms [4,11,14].

Although mutations in Fbxw7/hCdc4 are frequent events in diverse tumor types, including endometrial carcinomas, cholangiocarcinomas, colorectal cancer and T-cell acute lymphoblastic leukemia [4,7,11,15,16], mutations are uncommon or absent in other malignancies [4,17]. Thus, alternative mechanisms for inactivation of Fbxw7/hCdc4 are likely to exist. Downregulation of *Fbxw7/hCdc4 *expression has been reported in glioma [18], gastric cancer [19] and colorectal [20], but the mechanism(s) responsible for loss of expression of Fbxw7/hCdc4 in cancer is not known.

Epigenetic inactivation of TSGs by promoter hypermethylation is a frequent event during tumorigenesis [21,22]. In breast cancer, the most common malignant disease in women, hypermethylation of specific genes has been associated with the response to therapy, prognosis, invasiveness and metastasis [23,24]. Hypermethylation of TSGs in breast cancer is often associated with clinicopathological factors predicting poor prognosis and consequently serves as potential therapeutic targets for demethylating agents. However, recent data also indicate that methylation of specific TSGs can predict sensitivity to chemotherapy thus opening up the potential for DNA methylation as a biomarker to further individualize cancer treatment in the future [25].

In the present study, we examined the possibility that *FBXW7/hCDC4 *expression is epigenetically inactivated through promoter specific hypermethylation in breast cancer, a tumor type where *Fbxw7/hCdc4 *mutations are not commonly observed [4]. We also explored the possibility that aberrant promoter methylation associates with clinical parameters and overall survival in breast cancer. The results demonstrate that 51% of primary breast tumor specimens have a methylated *FBXW7/hCDC4-β *promoter with concomitant loss of *FBXW7/hCDC4-β *expression. Interestingly, although methylation associates with high-grade tumors, univariate and multivariate analysis suggest that *FBXW7/hCDC4-β *promoter methylation might be a favorable prognostic marker in breast cancer.

## Materials and methods

### Tumor specimens and clinicopathological features

A total of 161 primary breast tumor specimens from two breast cancer cohorts were included in this study. Among the 161 samples in which *FBXW7/hCDC4-β *promoter methylation was analysed, RNA was available from 139 samples, which was further processed for cDNA synthesis and *FBXW7/hCDC4-β *expression analysis as described below. A total of 68 cases of primary breast cancer were obtained from patients diagnosed at the Department of Obstetrics and Gynecology of the Innsbruck Medical University of Austria (cohort 1) and 93 samples were obtained from patients at the Department of Pathology of Uppsala University, Uppsala, Sweden (cohort 2). All patients underwent resection of the tumor during surgery and specimens were processed by pathologists at the affiliated hospital. Samples were snap frozen in liquid nitrogen and stored at -80°C until RNA and DNA extraction.

Cohort 1: Clinicopathologic features for this collection of samples have been previously reported [26]. Patients were diagnosed and operated on between 1990 and 2001 and the median age of the patients included in this study was 64 years (range 33 to 88). Patients were treated in compliance with the national recommendations at the time. Forty-one and 27 patients underwent a lumpectomy or a mastectomy, respectively. Thirty-eight patients received loco-regional radiation. Thirty-five patients received adjuvant combination chemotherapy CMF (cyclophosphamide, methotrexate and 5-fluorouracil), and 33 patients received adjuvant anti-hormonal therapy. All samples were collected during surgery in compliance with and approved by the Institutional Review Board and with informed consent from the patients. *p53 *was sequenced in tumor samples from cohort 1 using either genomic DNA or cDNA as a template with primers derived from intronic or gene specific sequences (primer sequences can be obtained from the authors upon request). PCR amplification and purification was performed as previously described [4,15] and sequenced at Eurofins MWG Operon (Eurofins MWG Operon, Ebersberg, Germany).

Cohort 2: Breast cancer patients were diagnosed and operated on between 1987 and 1989 at Uppsala University Hospital, Uppsala, Sweden. The median age for the patients used in this study was 63 years (range: 28 to 94). Clinicopathological data and treatment have been previously reported [27,28]. Briefly, patients were operated on and received postoperative radiotherapy. When adjuvant tamoxifen was given, some patients received this treatment as part of a randomized study comparing two versus five years, and chemotherapy, mostly CMF according to standards in those days [27,28]. Ethical permission was obtained from the ethical committee at Karolinska Institute and with informed consent from the patients. Data on *p53 *mutational status in this series of breast tumor specimens have been described [27].

Genomic DNA and RNA from fresh frozen tumor tissue were isolated as previously described [4,15]. RNA from various normal tissues was purchased from ABI (Applied Biosystems, Foster city, CA, USA). Normal breast tissue was obtained from reduction surgeries as well as from noncancerous tissue DNA extracted from paraffin-embedded breast cancer specimens. cDNA was synthesized from 2 μg of total RNA using Superscript First-strand Synthesis System (Invitrogen, Carlsbad, CA, USA).

### Cell lines, transfections and treatments

A total of 60 human cancer cell lines, originating from breast, brain, prostate, kidney, blood, cervix, lung, skin, bone and thyroid (See Additional file 1 for details), were analyzed for *FBXW7/hCDC4 *expression and promoter methylation. Cell lines were maintained and cultured according to American Type Culture Collection (ATCC) guidelines or as previously described [15,29]. All plasmid transfections were performed using LT1 transfection reagent (MIRUS, Madison, WI, USA), as recommended by the manufacturer's protocol. For experiments evaluating the effects of demethylation, cell lines maintained in appropriate media were treated with 5-aza-2'-deoxycytodine (5-aza-dC) (Sigma-Aldrich, St Louis, MO, USA) or DMSO for three to five days.

### Cloning

A 1.6 kb genomic region (-1293 bp to +309 bp from the transcription start site (TSS)) of the *FBXW7/hCDC4-β *isoform containing 18 CpG sites was amplified by PCR, cloned into the pSC-A vector (Stratagene, Santa Clara, CA, USA) and sequenced. The specific primers used for amplification of the *FBXW7/hCDC4-β *promoter, were as follows; FP1: 5'-GCC ATT TAC CAC CAT AGC AGA GAG TA-3', RP1: 5'-GCT ATG TGA TTG TGT GTG TAT GCC-3'. Two shorter versions of the promoter, containing 11 and 6 CpGs respectively, were also amplified using the following forward primers, FP2: 5'-AGA CTT ATT TGT GGA AAT GTT CCT TGC TA or FP3: 5'-GCA TTG CTG AAT CCT GGA CTG CAC C and the reverse primer, RP1. Each promoter region was subcloned into pGL3 vector (Promega, Madison, WI, USA) after KpnI and BglII restriction digestion (New England Biolabs, Ipswich, MA, USA). The resulting promoter constructs were termed pGL3hCDC4*β*-1.6, pGL3hCDC4*β*-0.8. and pGL3hCDC4*β*-0.6. The pRL-SV40 Renilla luciferase plasmid was obtained from Promega (Madison). PCR reactions were performed in a BioRad thermocycler (Techne, Burlington, NJ, USA).

### Luciferase assay

Luciferase activities in cell lysates from cells transfected with different pGL3 constructs and pRL-SV40 control plasmid were measured in a luminometer (VICTOR3, PerkinElmer, Waltham, MA, USA) using the Dual-Luciferase Reporter Assay System according to the manufacturer's protocol (Promega). Luciferase activities were quantified and fold change was averaged from at least three separate experiments performed in triplicates.

### DNA methylation analysis

Methylation of the *FBXW7/hCDC4-β *promoter was examined in cell lines, normal breast and primary tumors by several methods. Bisulfite-modified genomic DNA was prepared and CpG methylation was analysed by bisulfite-sequence analysis as previously described [30,31]. The methylation status of the complete *FBXW7/hCDC4-β *promoter was determined by sequence analysis of at least five individual clones from cell lines if not otherwise stated. For screening of relative methylation levels, the McrBc restriction enzyme (New England Biolabs) was used. McrBc recognize and cuts pairs of purine-methyl cytosines (recognition sequence R^m^C(N)_55-103_R^m^C) and subsequent PCR amplification of methylated DNA segments in comparison with an unmethylated segments thus denotes the methylation status of the region of interest. Briefly, 200 ng of genomic DNA was treated with or without 0.5 unit of McrBc enzyme for 1 hr at 37°C in the reaction buffer provided by the supplier. Each sample was heat inactivated and subsequently amplified by PCR using FP1 and RP1 primers. PCR amplification was performed with 100 ng DNA as template in the following conditions; two minutes denaturation and 30 cycles of amplification (94°C for 30 s, 64°C for 30 s, and 68°C for one minute) using Titanium™taq DNA polymerase (BD) (Becton dickinson, Franklin Lakes, NJ, USA). PCR products were resolved on agarose gels and band intensities were quantified by Image J software (ImageJ, U. S. National Institutes of Health, Bethesda, Maryland, USA) [32] The mean McrBc ratio (band intensity of McrBc digested DNA divided by undigested DNA) in unmethylated DNA samples obtained from normal breast tissue and tumor-derived cell lines (verified through bisulfite sequence analysis) was 0.808 with a standard deviation (SD) of 0.11. Test samples were judged as methylated if their McrBc ratio had a decreased value greater than 2 SD as compared to the unmethylated control samples. A McrBc methylation ratio of 0.6 was thus used as a cutoff. The McrBc results were also confirmed by restriction digestion of PCR products using Taq I and HpyCHIV enzymes (cuts methylated CG11 and CG18, respectively, data not shown). To verify the effect of promoter methylation on *FBXW7/hCDC4-β *expression, luciferase reporter constructs were methylated *in vitro *with Sss I methylase (New England Biolabs) as previously described [33]. Screening for methylation of *FBXW7/hCDC4-α *in primary tumor samples was carried out using McrBc restriction digestion analysis. The *FBXW7/hCDC4-α *promoter was amplified using primers; FA1: 5'-AGA CCC AGG AAG AGG AAA AGA GGA-3', RA1: 5'-TGG GTT GGT TCC CTT CCT CCT TC-3' and analysed for methylation as described above.

### Expression analysis

*FBXW7/hCDC4 *isoform-specific primers and TaqMan probes for quantitative real-time PCR analysis of different *FBXW7/hCDC4 *isoforms have been described [12]. The ^ΔΔ^Ct method of relative quantification was performed to determine relative mRNA expression in each sample. The relative expression level of each *FBXW7/hCDC4 *isoform was obtained by normalizing the expression of *FBXW7/hCDC4 *mRNA to *GAPDH *mRNA expression. Primers and conditions for semi-quantitative RT-PCR of *FBXW7/hCDC4 *isoforms have been described [29]. Amplification of *GAPDH *mRNA served as an internal control. Oestrogen and progesterone receptor (ER/PR) status was determined by immunohistochemistry as previously described [34].

### Statistical analysis

The association between patient clinicopathological characteristics (such as oestrogen receptor, progesterone receptor, lymph node involvement, grade, stage and age) and methylation status of the *FBXW7/hCDC4-β *isoform, in addition to *P53 *mutation, was determined using the Fisher's exact test. Differences in *FBXW7/hCDC4-β *expression between methylated and unmethylated groups were analysed by means of the (non-parametric) Wilcoxon-Mann-Whitney test. Univariate analyses of survival were conducted by use of the Kaplan-Meier method. Further, the risk of dying in women with methylation of the *FBXW7/hCDC4-β *isoform compared with women with unmethylated *FBXW7/hCDC4-β *isoform was modelled by use of multivariable proportional hazards (Cox) models, adjusted for possible confounders of survival such as age at diagnosis, date of primary tumour surgery, oestrogen receptor and progesterone receptor status, and *P53 *mutation. An arbitrary level of 5% statistical significance was used. Finally, data preparation and analysis was done using the SAS Statistical package, version 9.2 (SAS Institute Inc., Cary, NC, USA).

## Results

### Identification of promoter methylation and loss of *FBXW7/hCDC4-β *expression in tumor cell lines

**Figure 1:**
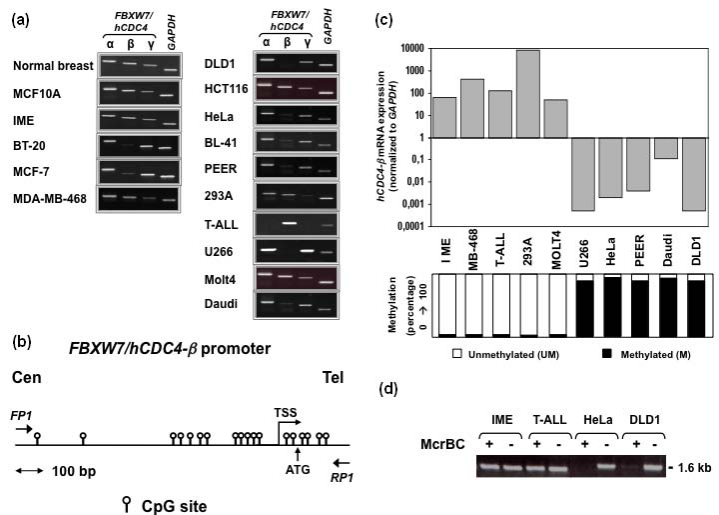
**Differential expression of *FBXW7/hCDC4-β *mRNA and promoter methylation in tumor cell lines**. **(a) **Semi-quantitative RT-PCR analysis of *FBXW7/hCDC4 *isoforms, *left*, in normal breast, immortalized mammary epithelial cells and breast cancer cell lines, *right*, in human cancer lines from different origins. *GAPDH *was amplified as an internal control. **(b) **Schematic map of the *FBXW7/hCDC4-β *promoter region around the transcription start site (TSS). CpG dinucleotides are depicted. PCR primers used for amplification of the promoter are indicated as arrows (FP1 and RP1). The translational start codon (ATG), centromere (Cen) and telomere (Tel) are marked. **(c) **real-time PCR showing the relationship between *FBXW7/hCDC4-β *expression (*top*) and promoter methylation (*bottom*); Methylation is presented as the percentage of methylated CpG sites based on bisulphate sequence analysis of genomic DNA from different cancer cell lines. The degrees of methylated cytosines or unmethylated cytosines are indicated by black or white boxes, respectively. **(d) **Representative gel analysis of PCR product (1.6 kb) from McrBc digested (+) genomic DNA in comparison with undigested (-) control DNA. Note the lack of amplification of McrBc digested DNA from methylated cell lines (HeLa and DLD1) in comparison to unmethylated cell lines (IME and T-ALL).

Mammals express three Fbxw7/hCdc4 splice variants designated α, β, and γ, each encoding a unique N-terminal protein sequence fused to 10 downstream exons, suggesting non-redundant functions as mentioned above. Semi-quantitative RT-PCR analysis of the different *FBXW7/hCDC4 *isoforms revealed substantial differential expression of the *FBXW7/hCDC4-β *transcript in specific cell lines from various tumor tissues (Figure 1a). The immortalized breast epithelial cell lines IME and MCF10, expressed high levels of *FBXW7/hCDC4-β *compared to some breast cancer cell lines with low or absent *FBXW7/hCDC4-β *expression (Figure 1a, *left*). This variation in mRNA expression was not generally observed for the *FBXW7/hCDC4-α *isoform, which was the most abundant and ubiquitously expressed *FBXW7/hCDC4 *transcript in most cell lines examined (Figure 1a).

As previously reported [35,36], the beta isoform was expressed at very high levels in tissue from normal brain (Figure S1 in Additional file 1). Significant expression was also observed in tissues from normal breast, ovary and cervix, compared to other tissues with low (spleen, thyroid, liver) or absent (skeletal muscle) *FBXW7/hCDC4-β *expression (Figure S1 in Additional file 1). To examine whether loss of *FBXW7/hCDC4-β *expression correlated with hypermethylation of its promoter, we first examined the sequence 1.3 kb upstream and 0.3 kb downstream of the transcription initiation start site in *FBXW7/hCDC4-β*. Eighteen CpG sites were distributed throughout this region (Figure 1b). To this end, we used bisulfite sequence analysis to determine the methylation status of each of these CpGs in five different cell lines with low/absent *FBXW7/hCDC4-β *expression (HeLa, U266, PEER, Daudi and DLD1) and five cell lines with high expression (IME, MB-468, T-ALL, 293A and MOLT4). Regarding the methylation status, cell lines were found to fall into two distinct groups; one demonstrating methylation of the majority of CpGs (90 to 100%) correlating with low expression, and the other exhibiting mostly unmethylated CpGs and showing high expression (Figure 1c). Screening for methylation was also carried out using the restriction enzyme McrBc, a methylation specific endonuclease, which cuts DNA containing 5-methylcytosine in the context of a second, arbitrarily spaced methylcytosine, and does not cleave unmethylated DNA. As shown in Figure 1d, screening for methylation of this region with the McrBc enzyme recapitulated the methylation results obtained by bisulphate sequence analysis.

**Figure 2:**
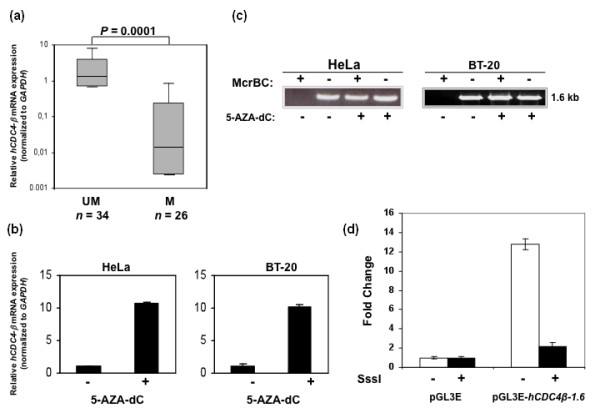
**Inverse correlation between *FBXW7/hCDC4-β *mRNA expression and promoter methylation in tumor cell lines**. **(a) **Cancer cell lines were divided into two groups based on the ratio of band intensity of McrBc digested cell line DNA divided by undigested DNA. The expression levels were lower in the methylated (M) group than in the unmethylated (UM) group (*P *= 0.0001). Horizontal lines, median expression value of each group. *P *value was calculated using the nonparametric Mann-Whitney *U *test. **(b) **5-aza-dC treatment upregulate *FBXW7/hCDC4-β *mRNA levels in methylated cell lines. Mean ± SD. **(c) **5-aza-dC demethylate the *FBXW7/hCDC4-β *promoter as evident by restoration of PCR amplification of McrBc digested DNA. **(d) **pGL3E luciferase reporter assay without or with *FBXW7/hCDC4-β *promoter region in vitro methylated using SssI enzyme (filled bars). Relative fold change in luciferase activity +/- SEM is shown.

Assessing methylation by McrBc digestion in 50 additional cell lines from various tissues (Table S1 in Additional file 2) demonstrated methylation in 26 of 60 (43%). *FBXW7/hCDC4-β *expression was next analyzed by real time reverse transcription PCR. A significant difference in expression between methylated and unmethylated cell lines (Figure 2a, *P *= 0.0001) was found with lower expression in methylated (median expression = 0.02, range; 0 to 0.9) compared to unmethylated cell lines (median expression = 1.6, range; 0 to 8.1). No correlation between methylation of the *FBXW7/hCDC4-β *promoter and expression of *FBXW7/hCDC4-α *mRNA was found (data not shown).

To establish a direct link between methylation and silencing of *FBXW7/hCDC4-β *expression we treated HeLa, BT-20, U2OS and BT-474 cells with the DNA methylation inhibitor 5-aza-dC. As can be seen in Figure 2b, 5-aza-dC treatment increased *FBXW7/hCDC4-β *mRNA expression levels in methylated cell lines (HeLa and BT-20), with an initial low expression, in contrast to the unchanged high *FBXW7/hCDC4-β *levels of unmethylated cell lines (U2OS and BT-474) (Figure S2A in Additional file 3). Demethylation of the promoter upon 5-aza-dC treatment was confirmed by McrBc digestion (Figure 2c) and sequencing of bisulfite treated DNA (data not shown). As a control, *FBXW7/hCDC4-α *mRNA expression was measured after 5-aza-dC treatment. No significant increase of *FBXW7/hCDC4-α *levels was observed in any of the cell lines examined (Figure S2B in Additional file 4 and data not shown).

To confirm that the *FBXW7/hCDC4-β *promoter region possesses transcriptional activity, we performed luciferase reporter assays with the 1.6 kb genomic region covering all 18 CpGs (Figure 1b) and two shorter promoter regions (0.8 and 0.6 kb), being deletion constructs of the 1.6 kb region mentioned above. Robust reporter activity was observed in cell lines of different origins with all three constructs, albeit a reduced activity was observed with the shorter promoter constructs (data not shown). To further evaluate the effect of methylation on promoter activity, we next performed reporter assays using transient transfection of constructs where the *FBXW7/hCDC4-β p*romoter was methylated *in vitro *using the Sss I methylase as previously described [33]. As shown in Figure 2d, *in vitro *methylation suppressed reporter activity confirming that methylation of the promoter abrogates *FBXW7/hCDC4-β *expression.

Together, these data demonstrate that CpG-methylation correlates with loss of *FBXW7/hCDC4-β *expression in tumor cell lines. These data also indicate that methylation could be a significant factor in regulating *FBXW7/hCDC4-β *expression in some malignancies, including breast cancer.

### *FBXW7/hCDC4-β *promoter methylation in primary breast cancer specimens

**Figure 3:**
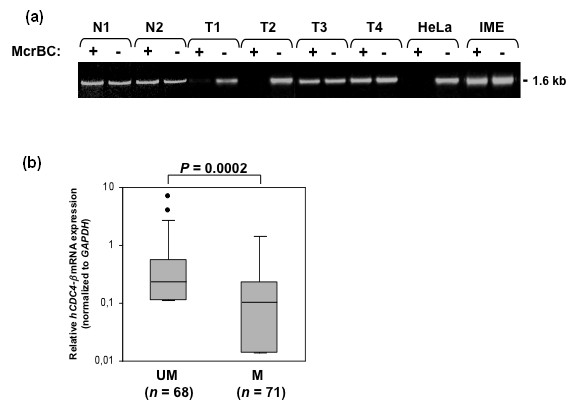
**Inverse correlation between *FBXW7/hCDC4-β *mRNA expression and promoter methylation in primary breast tumor specimens**. **(a) **Representative analysis of *FBXW7/hCDC4-β *promoter methylation in normal breast (N), breast tumor specimens (T), immortalized breast epithelial cells (IME) and a cancer cell line (HeLa). Genomic DNA was incubated with (+) or without (-) McrBc enzyme and the promoter was PCR amplified (1.6 kb). The cleavage pattern indicates differential methylation in tumor samples (T1, T2; methylated and T3, T4; unmethylated) and a lack of methylation normal breast (N1, N2). **(b) **Relationship between *FBXW7/hCDC4-β *mRNA expression levels and promoter methylation in primary breast tumors. Samples were stratified by relative methylation status (UM; unmethylated group, *N *= 68, M; methylated group, *N *= 71, see materials and methods for classification of methylation). *FBXW7/hCDC4-β *expression levels (normalized to *GAPDH *mRNA) were lower in the methylated group compared to the unmethylated group (Mann-Whitney *U *test, *P *= 0.0002). Bars from each box extend to largest and smallest expression levels. Outliers are plotted as asterisks (*).

Based on the results above, we next explored the occurrence and role of *FBXW7/hCDC4-β *methylation in primary breast cancer specimens. To exclude the possibility that methylation detection is affected by contaminating noncancerous cells, we first analysed *FBXW7/hCDC4-β *promoter methylation in normal breast tissues (obtained from normal breast reductions) by McrBc digestion (Figure 3a) and bisulfite sequence analysis (data not shown). Importantly, methylation was absent in normal breast tissues as well as in noncancerous tissue DNA extracted from a paraffin-embedded breast cancer specimen (Figure 3a and data not shown). We next analyzed *FBXW7/hCDC4-β *methylation in two cohorts of breast cancer specimens in which RNA was available and *FBXW7/hCDC4 *mRNA expression could be analysed. A total of 71 out of 139 (51%) patient samples showed methylation of the promoter as defined by McrBc digestion, compared to its undigested control (Figure 3a). Tumor specimens were classified as low or high methylation (see Materials and methods for details on quantification and classification of promoter methylation), divided into two groups and compared with the mRNA expression levels of *FBXW7/hCDC4-β*. Similar to the results in tumor cell lines, a statistically significant difference was found between the groups (Figure 3b, *P *= 0.0002). The methylated group showed significantly lower expression of *FBXW7/hCDC4-β *(median expression = 0.13, range; 0 to 1.43) as compared to the unmethylated group, which had higher expression levels (median expression = 0.30, range; 0 to 8.83).

These results demonstrate that there is a significant inverse correlation between promoter methylation and *FBXW7/hCDC4-β *expression in primary breast cancer specimens.

### Correlation of *FBXW7/hCDC4-β *methylation with clinicopathological characteristics

Data on methylation status and clinicopathological parameters were available for a total of 161 tumor specimens (68 cases from cohort 1 and 93 cases from cohort 2). The associations between *FBXW7/hCDC4-β *methylation and clinicopathological features are summarized in Table 1. Methylation was significantly associated with high-grade tumors (*P *= 0.017) and a trend towards an association with estrogen receptor-negative tumors was also observed in the methylated group (Table 1). No significant associations were observed between methylation of *FBXW7/hCDC4-β *with age, stage and lymph node status (Table 1) or with p53 mutational status (Table S2 in Additional file 5). As previously reported [27], p53 mutation was associated with high-grade and receptor-negative (ER and PR) tumors (Table S2 in Additional file 5).

**Table 1:** Association of FBXW7/hCDC4-β promoter methylation in 161 primary breast cancer patients with clinicopathological features

	UM	(%)	M	(%)	*P*
**Age**					
≤67	37	(45.1)	45	(54.9)	0.27
>67	43	(54.4)	36	(45.6)	
**ER**					
Negative	17	(37.7)	28	(62.3)	0.08
Positive	61	(54)	52	(46)	
Unknown	2		1		
**PR**					
Negative	17	(40)	25	(60)	0.21
Positive	61	(52.5)	55	(47.5)	
Unknown	2		1		
**LN**					
Negative	41	(50.6)	40	(49.4)	0.75
Positive	36	(47)	40	(53)	
Unknown	3		1		
**Grade**					
I/II	64	(55.1)	52	(44.9)	0.017
III	13	(32.5)	27	(67.5)	
Unknown	3		2		
**Stage**					
I/II	58	(52.2)	53	(47.8)	0.18
III	9	(36)	16	(64)	
Unknown	12		12		

* Fisher's exact test. UM, unmethylated; M, methylated; ER, estrogen receptor; PR, progesterone receptor; LN, lymph node.

### Correlation of *FBXW7/hCDC4-β *methylation and prognosis

**Figure 4:**
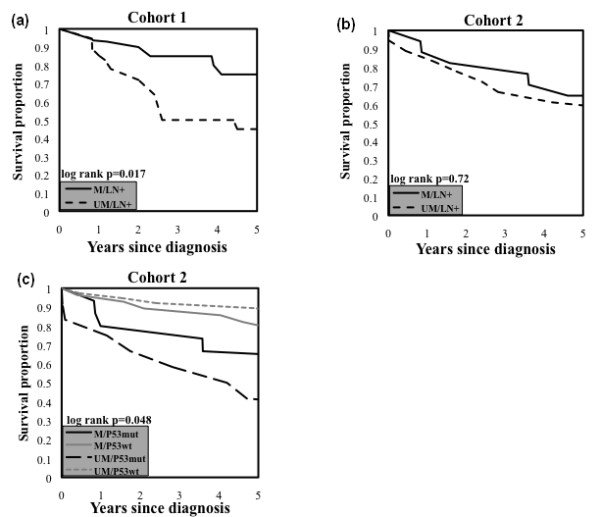
**Kaplan-Meier analysis of overall survival in specific patient subgroups**. **(a) **Overall survival rate for patients with methylated *FBXW7/hCDC4-β *promoter was significantly higher than that for patients in the unmethylated group in lymph node positive patients from cohort 1 (log rank test, *P *= 0.017). **(b) **Overall survival for patients from cohort 2 showed a similar trend as patients in cohort 1 but was not statistically significant (log rank test, *P *= 0.72). **(c) **Patients with methylated *FBXW7/hCDC4-β *promoter and p53 mutation have improved survival compared to patients with p53 mutation and unmethylated *FBXW7/hCDC4-β *promoter in cohort 2 (log rank test, *P *= 0.048).

To evaluate whether methylation of *FBXW7/hCDC4-β *was an independent prognostic factor in breast cancer, we examined the significance of methylation using multivariate analysis, including adjustment for other factors known to be associated with clinical outcome. As the cohorts from the two centers were treated during different time periods, and thus partly using different treatment protocols, this outcome analysis was performed separately for the two groups. This also enabled a validation of the findings in the two independent cohorts. In both cohorts, methylation of *FBXW7/hCDC4-β *was found to be associated with an approximately 50% decreased risk of death (cohort 1 hazard ratio 0.53 (0.23 to 1.23) and cohort 2 (HR) 0.50 (95% CI 0.23 to 1.08), Table 2). We also performed log rank analysis of Kaplan-Meier curves for overall survival in defined prognostic subgroups. Methylation was associated with a significantly improved overall survival in patients with lymph node metastasis, in cohort 1 (Figure 4a, *P *= 0.017). In this cohort, patients with ER receptor-negative tumors additionally demonstrated a clear trend, although not statistically significant (*P *= 0.111, data not shown), for improved survival in methylated tumors. In line with the data from cohort 1, the overall survival in women with lymph node-positive tumors from cohort 2 was higher in the group whose tumors demonstrated a methylated *FBXW7/hCDC4-β *promoter, compared to women with an unmethylated promoter (Figure 4b). However, this difference was not statistically significant, possibly due to the smaller number of patients in this subgroup from cohort 2 (*n *= 35 out of 93), in comparison with cohort 1 (*n *= 41 out 68). No clear conclusions could be drawn from cohort 2 regarding the prognostic significance for *FBXW7/hCDC4-β *methylation in the ER-negative group, due to the limited number of patients (*n *= 13). Methylation was also associated with a significantly improved overall survival in the p53 mutated subgroup in cohort 2 (Figure 4c, *P *= 0.048). Thus, in two subgroups with known adverse prognostic features (that is, patients with lymph node metastasis and patients whose tumors harbored p53 mutation) *FBXW7/hCDC4-β *methylation was found to be associated with improved survival. No association between survival and methylation of *FBXW7/hCDC4-β *was found in patients with lymph node negative tumors (Figure S3 in Additional file 6). No clear conclusions could be drawn from cohort 1 regarding the prognostic significance for patients with p53 mutation and *FBXW7/hCDC4-β *methylation due to the limited number of patients and events in the p53 mutated/*FBXW7/hCDC4-β *methylated subgroup.

**Table 2:** Methylation status and overall survival in primary breast cancer patients using Cox proportional hazard model

	Patients Φ	(%)	Deaths	HR	95%CI*	*P*
**Cohort 1**						
UM	30	(44.1)	14	1.0(ref)		
M	38	(55.9)	12	0.53	0.23 to 1.23	0.14
**Cohort 2**						
UM	50	(53.8)	21	1.0(ref)		
M	43	(46.2)	28	0.50	0.23 to 1.08	0.08

^Φ ^Cohort 1 (*n *= 68) and Cohort 2 (*n *= 93).

* Adjusted for age, period of diagnosis, ER, PR and p53 status. HR, hazard ratio; UM, unmethylated; M, methylated.

## Discussion

F-box proteins are substrate recognition subunits of the SCF ubiquitin ligases with well-established functions in cell cycle control and tumor development [37,38]. We recently performed a comprehensive analysis of primary human tumors and showed mutations in the F-box gene *FBXW7/hCDC4 *to occur with an overall frequency of 6% in diverse tumor types [4]. *FBXW7/hCDC4 *mutations were first identified in breast cancer and ovarian cell lines [14,35]. However, analysis of more than 151 primary breast tumor specimens showed that *FBXW7/hCDC4 *mutations are rare in this malignancy [4].

Several studies have reported reduction in *FBXW7/hCDC4 *copy number due to deletion of chromosome 4q [6,39]. However, loss of *FBXW7/hCDC4 *expression in cancer can occur without genetic alteration [20], implicating a possible role for epigenetic silencing of *FBXW7/hCDC4*.

In this study, we report for the first time promoter specific methylation of the *FBXW7/hCDC4-β *isoform in primary breast tumors. Two independent cohorts of primary breast tumors were analyzed and promoter hypermethylation was found to correlate with downregulation of *FBXW7/hCDC4-β *mRNA expression (Figure 3b). Moreover, using DNA methylation inhibitors in intact cells as well as *in vitro *methylation of promoter constructs in reporter assays, we demonstrate that *FBXW7/hCDC4-β *expression is regulated by promoter hypermethylation (Figure 2). Loss of *FBXW7/hCDC4 *expression is unlikely to result from allelic imbalances of the 4q31 locus, as examination of >140 primary breast tumors using Representational Oligonucleotide Microarray Analysis (ROMA [40]), showed that *FBXW7/hCDC4 *is hemizygously deleted in less than 20% of primary breast tumors (data not shown, A. Zetterberg and P Lundin, personal communication).

In breast cancer ER/PR and HER-2/neu represent the few established molecular markers used both for prognostication as well as to predict the response to tamoxifen and herceptin, respectively [41,42]. Aberrant DNA methylation is a common event in cancer and a wealth of recent data indicates that altered methylation of TSGs in breast cancer is frequent, and that determination of such events can provide prognostic as well as predictive information [23,24,43]. However, there is still an urgent need for discovery of novel prognostic factors and predictive biomarkers that give pre-treatment information on the efficacy of adjuvant chemotherapy, which is commonly applied to many patients with primary breast cancer.

Methylation of TSGs often associates with adverse clinical factors [44-46]. In line with this, in this study, *FBXW7/hCDC4-β *promoter methylation significantly was associated with high-grade tumors and also occurred frequently in ER-negative tumors (Table 1). Interestingly, despite this, multivariate survival analysis demonstrated an association between methylation of *FBXW7/hCDC4-β *and decreased risk of death (Table 2). As mentioned above, the differences between the cohorts prohibited us from combining the data. It will thus be important to validate these findings in larger clinical cohorts.

Interestingly, sub-group analysis revealed that *FBXW7/hCDC4-β *methylation identifies patients with a significantly improved prognosis among patients whose tumors demonstrated the adverse features of lymph node metastasis and p53 mutation, respectively (Figure 4). This somewhat parallels previous findings from our group [15] and others [47] in T-cell acute lymphocytic leukemia (T-ALL), an ALL sub-group with relatively poor prognosis. In this malignancy, mutational inactivation of *FBXW7/hCDC4 *in combination with *NOTCH1 *mutations predicts a favorable outcome [15,47].

One may speculate why methylation of *FBXW7/hCDC4-β *in breast cancer associates with improved outcome. One reason may be that methylated tumors have a biologically less aggressive behavior. This seems less likely, in light of our findings that these tumors are overrepresented in subgroups of patients with high-grade tumors, and possibly ER-negativity. In line with this, we have preliminary data demonstrating an association between *FBXW7/hCDC4-β *methylation and high expression of the proliferation marker PCNA in breast cancer (correlation coefficient r = 0.313, *P *= 0.022 (*n *= 52, cohort 1)). Thus, a more likely possibility is that tumors with inactivated *FBXW7/hCDC4-β *may be more responsive to the given adjuvant treatment. This could also explain the greater impact of *FBXW7/hCDC4-β *methylation on survival in cohort 1, compared to cohort 2, as a higher proportion of patients in cohort 1 were exposed to adjuvant polychemotherapy (see materials and methods and 27). Furthermore, a link between *FBXW7/hCDC4-β *methylation and a tendency for increased survival was found in patients receiving chemotherapy and/or irradiation (Table S3 in Additional file 7), as analyzed in cohort 1.

*FBXW7/hCDC4 *function has been directly linked to cell cycle checkpoint controls, its inactivation resulting in improper M phase progression, formation of micronuclei and chromosomal instability [7]. Indeed, Finkin *et al*. hypothesized that loss of *FBXW7/hCDC4 *might have important effects on how cells respond to chemotherapeutic drugs, and showed that exposure of cells lacking *FBXW7/hCDC4 *to spindle toxins, such as taxol, commonly used in breast cancer treatment, renders cells more susceptible to endoreduplication and polyploidy [48].

The recent advances and understanding of how cell cycle checkpoints and DNA repair pathways respond to chemotherapy-induced DNA damage has opened up unprecedented opportunities for improved development of personalized treatment [49]. This is for instance exemplified in a recent elegant study that demonstrated a synthetic lethal interaction between ATM and p53 in response to cytotoxic drugs [50]. Remarkably, tumors defective for both ATM and p53 were more prone to drug induced cell killing and tumors with functional ATM but non-functional p53 could be sensitized by pharmacologic suppression of ATM signaling [50,51]. Similar results have been reported for other TSGs involved in controlling genomic stability, such as the inhibition of PARP-1 in BRCA1/2 deficient breast and ovarian tumors which selectively kills the cancer cells while sparing normal cells with a functional repair pathway [52]. Together, these findings highlight the importance for a detailed knowledge of genetic defects in tumors to identify those patients who will most likely respond to specific genotoxic drugs.

To date, defined Fbxw7/hCdc4 substrates are nuclear proteins (that is, c-Myc, cyclin E) degraded by the Fbxw7/hCdc4-*α *and/or Fbxw7/hCdc4-*γ *isoforms [1,2], whereas Fbxw7/hCdc4-*β *specific substrates still await discovery. Fbxw7/hCdc4-*β *localizes to the cytoplasm and could potentially target a pro-apoptotic substrate(s) or a protein(s) involved in DNA damage signaling that when stabilized sensitize tumor cells to chemotherapeutic drugs. Interestingly, Mao *et al*. recently reported that Fbxw7/hCdc4 targets the mammalian target of rapamycin (mTor) for degradation [53]. The Fbxw7/hCdc4 isoform responsible for mTor degradation was not defined in this study, but it's noteworthy that mTor localizes to the cytosol and that breast cancer cells with loss of Fbxw7/hCdc4 were shown to be more sensitive to mTor inhibitors [53].

We have just begun to understand the complex interplay between *FBXW7/hCDC4*, its target oncoproteins and other critical cancer genes. For example, p53 is a direct transcriptional regulator of *FBXW7/hCDC4 *expression [10]. However, the functional relationship between these TSGs is still unclear and *FBXW7/hCDC4 *has also been suggested to act upstream of p53 [48]. Furthermore, a differential relationship with the various *FBXW7/hCDC4 *isoforms is possible. A recent study in gastric cancer revealed that patient samples with p53 mutations had lower *FBXW7/hCDC4-α *mRNA levels and those patients also had a poor prognosis compared with the other subgroups [19]. In line with these data, in our analysis of breast cancer specimens, we also found a statistically significant correlation (*P *= 0.0002) between low *FBXW7/hCDC4-α *expression and p53 mutation. To exclude the possibility that downregulation of *FBXW7/hCDC4-α *expression is due to promoter methylation, we have analysed the *FBXW7/hCDC4-α *CpG island for methylation. Methylation was not observed in any of the primary breast tumor specimens analysed, independently of *FBXW7/hCDC4-α *mRNA levels, indicating that p53 mutational status is major decisive factor in the regulation of *FBXW7/hCDC4-α *expression in breast cancer (manuscript in preparation). Interestingly, no significant association between the expression (or methylation) of *FBXW7/hCDC4-β *and p53 mutational status (Table S2 in Additional file 5 and data not shown) was found, and as mentioned above, methylation and loss of expression of *FBXW7/hCDC4-β *was instead linked to increased survival in the p53 mutated group. Thus, although *FBXW7/hCDC4-β *is a known transcriptional target of p53 [10], methylation of the promoter likely predominates in transcriptional suppression of *FBXW7/hCDC4-β *mRNA expression. In summary, we have identified a previously not recognized mechanism for inactivation of *FBXW7/hCDC4 *expression, namely promoter specific methylation with potential prognostic significance in breast cancer. These data suggest that methylation of the *FBXW7/hCDC4-β *promoter predicts a favorable prognosis in breast cancer, particular in specific patient subgroups, despite the association of *FBXW7/hCDC4-β *methylation with adverse clinical parameters. Further studies will be required to validate whether *FBXW7/hCDC4-β *methylation could serve as a biomarker for sensitivity to chemotherapy in breast cancer.

## Conclusions

This study provides new insights into the causes and consequences of *FBXW7/hCDC4 *inactivation in breast cancer. The *FBXW7/hCDC4-β *promoter is methylated in approximately 50% of primary breast tumors and methylation correlates with loss of *FBXW7/hCDC4-β *expression. Furthermore, promoter methylation predicts a favorable prognosis in breast cancer, particularly in specific patient subgroups, despite the association of *FBXW7/hCDC4-β *methylation with adverse clinical parameters. The role of *FBXW7/hCDC4-β *methylation in breast cancer progression and its potential function as a novel biomarker for sensitivity to chemotherapy in breast cancer needs further investigation.

## Abbreviations

CPD: CDC4 phosphodegron; ER: estrogen receptor; HR: hazard ratio; LN: lymph node; M: methylated; PCR: polymerase chain reaction; PR: progesterone receptor; QRT-PCR: quantitative real-time PCR; RT-PCR: reverse transcriptase PCR; SCF: (SKP1/CUL1/F-box); SD: standard deviation; TSG: tumor suppressor gene; UM: unmethylated

## Competing interests

The authors declare that they have no competing interests.

## Authors' contributions

SA performed laboratory experiments, data analysis and wrote the manuscript. MC assisted in methylation analysis. OS, SA and DG initiated and designed the study. OS, DG, CS and JB were involved in experimental design and preparation of the manuscript. JB and MW were responsible for the patient cohorts. LL, MW and SA performed statistical analyses. All authors have read and approved the final manuscript.

## Supplementary Material

Additional file 1 **Supplemental Figure S1**. *FBXW7/hCDC4-β *expression levels were examined by real-time PCR (and normalized to *GAPDH *mRNA levels) in a panel of different normal tissues. *FBXW7/hCDC4-β *was differentially expressed between tissues, with the highest expression in brain and moderate expression in cervix, ovary and breast. Low or no detectable expression was found in spleen, thyroid, liver and skeletal muscle, respectively. Click here for file

Additional file 2 **Supplemental Table S1**. Panel of cell lines analyzed for *FBXW7/hCDC4-β *promoter methylation and expression. Click here for file

Additional file 3 **Supplemental Figure S2A**. *FBXW7/hCDC4-β *expression levels in unmethylated cell lines BT-474 and U2OS after 5-aza-dC treatment. Mean ± SD. Click here for file

Additional file 4 **Supplemental Figure S2B**. *FBXW7/hCDC4-α *expression levels in methylated cell lines HeLa and BT-20 after 5-aza-dC treatment. Mean ± SD. Click here for file

Additional file 5 **Supplemental Table S2**. Association of p53 mutation, clinicopathological features and *FBXW7/hCDC4-β *methylation in primary breast cancer patients. Click here for file

Additional file 6 **Supplemental Figure S3**. Kaplan-Meier analysis of overall survival in lymph node negative patients from cohort 1 and cohort 2. Click here for file

Additional file 7 **Supplemental Table S3**. Association between *FBXW7/hCDC4-β *methylation status and treatment outcome in breast cancer patients (cohort 1). Click here for file
