# Supplementary figures and images for: Inactivation of FBXW7/hCDC4-β expression by promoter hypermethylation is associated with favorable prognosis in primary breast cancer
Source: Breast Cancer Res. 2010 Dec 1;12(6):R105. doi: 10.1186/bcr2788 (PMC3046450; doi:10.1186/bcr2788)

Supplementary Figure 1

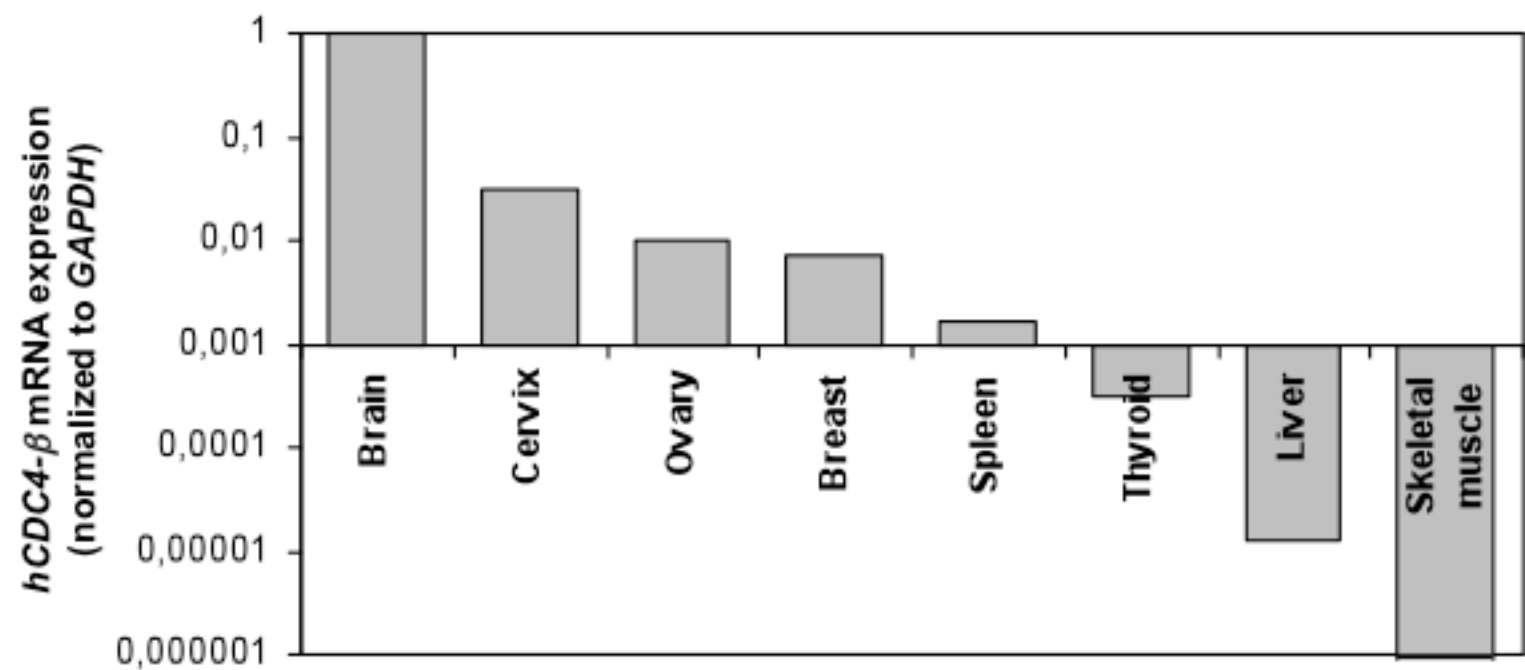

Supplement: Additional file 1 — Supplemental Figure S1. FBXW7/hCDC4-β expression levels were examined by real-time PCR (and normalized to GAPDH mRNA levels) in a panel of different normal tissues. FBXW7/hCDC4-β was differentially expressed between tissues, with the highest expression in brain and moderate expression in cervix, ovary and breast. Low or no detectable expression was found in spleen, thyroid, liver and skeletal muscle, respectively. [file bcr2788-S1.PDF]

## Supplementary Figure 2

A

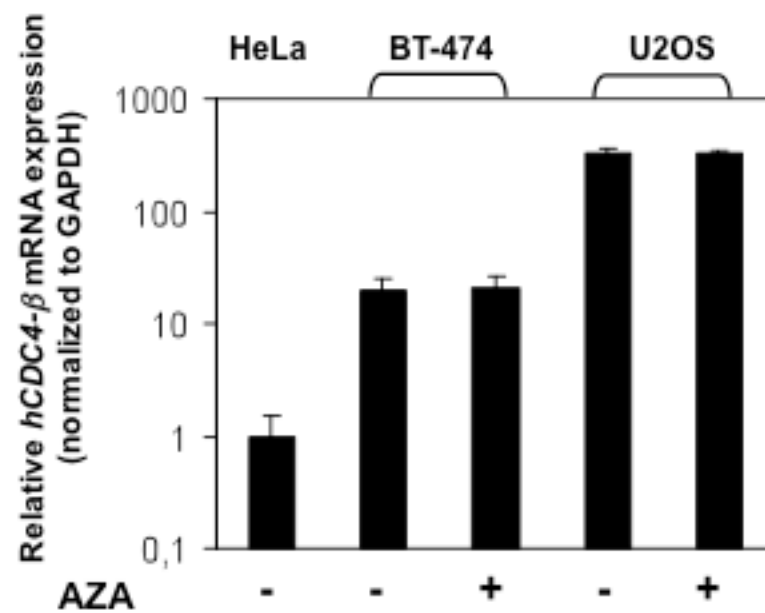

Supplement: Additional file 3 — Supplemental Figure S2A. FBXW7/hCDC4-β expression levels in unmethylated cell lines BT-474 and U2OS after 5-aza-dC treatment. Mean ± SD. [file bcr2788-S3.PDF]

## Supplementary Figure 2

**B**

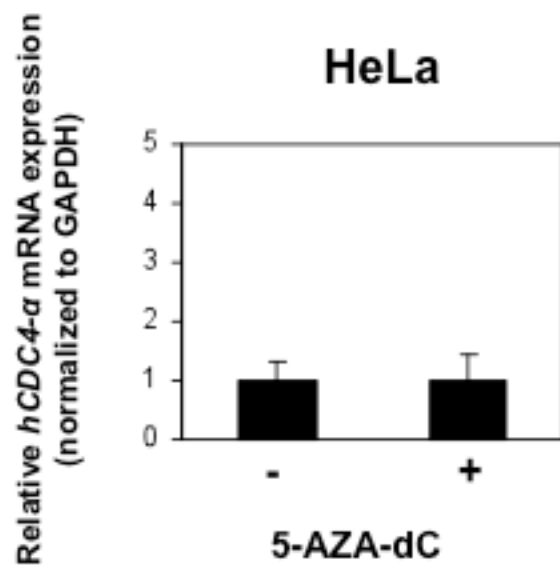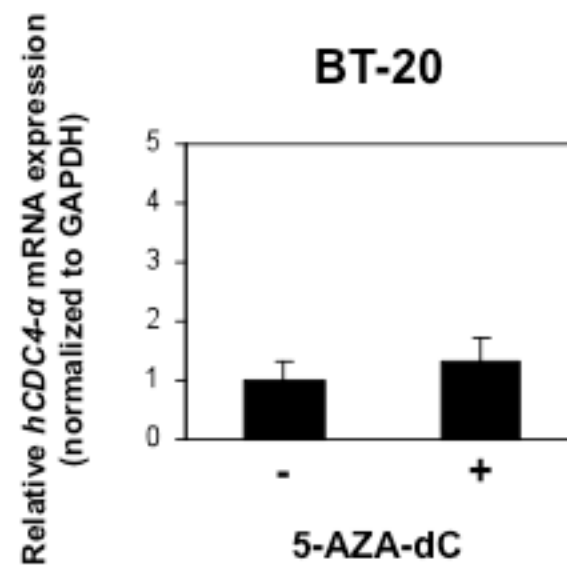

Supplement: Additional file 4 — Supplemental Figure S2B. FBXW7/hCDC4-α expression levels in methylated cell lines HeLa and BT-20 after 5-aza-dC treatment. Mean ± SD. [file bcr2788-S4.PDF]

**Supplementary Figure 3**

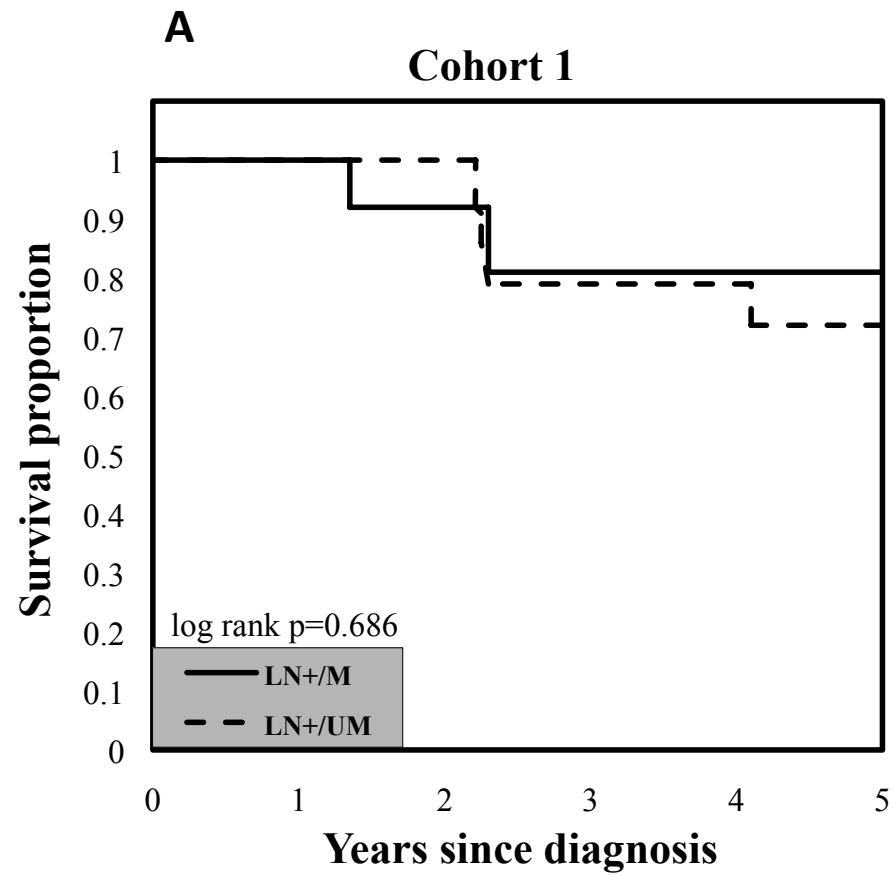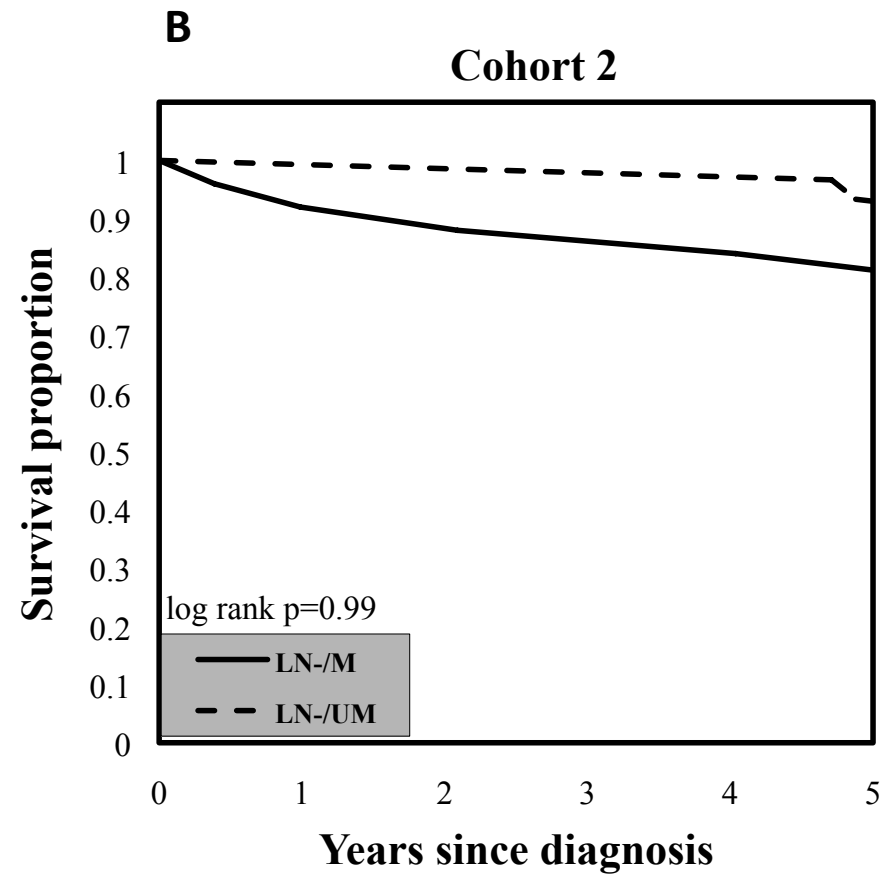

Supplement: Additional file 6 — Supplemental Figure S3. Kaplan-Meier analysis of overall survival in lymph node negative patients from cohort 1 and cohort 2. [file bcr2788-S6.PDF]
