# Supplementary material for: Inactivation of FBXW7/hCDC4-β expression by promoter hypermethylation is associated with favorable prognosis in primary breast cancer
Source: Breast Cancer Res. 2010 Dec 1;12(6):R105. doi: 10.1186/bcr2788 (PMC3046450; doi:10.1186/bcr2788)
Supplement: Additional file 2 — Supplemental Table S1. Panel of cell lines analyzed for FBXW7/hCDC4-β promoter methylation and expression. [file bcr2788-S2.PDF]

**Supplementary Table 1: Cell lines**	
**Tissue**	**Cell line**
Breast	MCF7, BT20, T47D, IME, SUM159PT, MDA-MB-435, SKBR, CAMA, SUM-BT-549, BT-474, ZR75, SUM PT-149, MDA-MB-468, MDA-MB-175
Brain	U343, U87, U251, U178-MG, U1240-MG, U563-MG
Blood	MOLT 4, JURKAT, PEER, CEM, Daudi, P12, H9, DND-41, JM, T-ALL, RS4, BM-T-ALL, SIL, HUT-78, U266, BL-41, 183E95
Lung	U1285, U1752, H82, U1810
Skin	OCM1, OCM8, OCM3, OCM9, SKMEL-28
Colon	DLD-1, HCT-116
Bone	U2OS, Saos2
Prostate	BU-145
Cervix	HeLa
Thyroid	KAT-4
Kidney	293A
