# Supplementary material for: Inactivation of FBXW7/hCDC4-β expression by promoter hypermethylation is associated with favorable prognosis in primary breast cancer
Source: Breast Cancer Res. 2010 Dec 1;12(6):R105. doi: 10.1186/bcr2788 (PMC3046450; doi:10.1186/bcr2788)
Supplement: Additional file 5 — Supplemental Table S2. Association of p53 mutation, clinicopathological features and FBXW7/hCDC4-β methylation in primary breast cancer patients. [file bcr2788-S5.DOC]

**Table S2. Association of *p53*** mutational status with clinicopathological features

|  | UM | (%) | M | (%) | *p* |
| --- | --- | --- | --- | --- | --- |
| **Age** |  |  |  |  |  |
| ≤67 | 62 | (74.6) | 21 | (25.4) | 0.71 |
| >67 | 61 | (77.2) | 18 | (22.8) |  |
| **Methylation** |  |  |  |  |  |
| UM | 63 | (78.7) | 17 | (21.3) | 0.46 |
| M | 59 | (72.8) | 22 | (27.2) |  |
| **ER** |  |  |  |  |  |
| Negative | 27 | (58.8) | 19 | (41.4) | 0.001 |
| Positive | 94 | (83.1) | 19 | (16.9) |  |
| Unknown | 2 |  | 1 |  |  |
| **PR** |  |  |  |  |  |
| Negative | 28 | (65.1) | 15 | (34.9) | 0.05 |
| Positive | 93 | (80) | 23 | (20) |  |
| Unknown | 2 |  | 1 |  |  |
| **LN** |  |  |  |  |  |
| Negative | 66 | (80) | 16 | (20) | 0.26 |
| Positive | 55 | (72.3) | 21 | (27.7) |  |
| Unknown | 2 |  | 2 |  |  |
| **Grade** |  |  |  |  |  |
| I/II | 104 | (88.8) | 13 | (11.2) | <0.0001 |
| III | 15 | (37.5) | 25 | (62.5) |  |
| Unknown | 4 |  | 1 |  |  |
| **Stage** |  |  |  |  |  |
| I/II | 89 | (79.4) | 23 | (20.6) | 0.78 |
| III | 19 | (76) | 6 | (24) |  |
| Unknown | 15 |  | 10 |  |  |

- Fisher’s exact test. UM, unmethylated; M, methylated; ER, estrogen receptor; PR, progesterone receptor;

LN, lymph node.
