# Supplementary material for: Inactivation of FBXW7/hCDC4-β expression by promoter hypermethylation is associated with favorable prognosis in primary breast cancer
Source: Breast Cancer Res. 2010 Dec 1;12(6):R105. doi: 10.1186/bcr2788 (PMC3046450; doi:10.1186/bcr2788)
Supplement: Additional file 7 — Supplemental Table S3. Association between FBXW7/hCDC4-β methylation status and treatment outcome in breast cancer patients (cohort 1). [file bcr2788-S7.DOC]

|  | **Methylated** | | **Unmethylated** | |  |
| --- | --- | --- | --- | --- | --- |
| **Therapy** | **Alive** | **Dead** | **Alive** | **Dead** | ***p* Value** |
| **Chemotherapy** | 18 | 6 | 8 | 3 | 0.2 |
| **Radiation** | 18 | 6 | 5 | 9 | 0.03 |
| **Radiation/ Chemotherapy** | 22 | 11 | 9 | 12 | 0.07 |

**Table 3. Association between methylation status of hCDC4 β promoter and treatment outcome in breast cancer patients**
